# Current State of Target Treatment in BRAF Mutated Melanoma

**DOI:** 10.3389/fmolb.2020.00154

**Published:** 2020-07-14

**Authors:** Enrica Teresa Tanda, Irene Vanni, Andrea Boutros, Virginia Andreotti, William Bruno, Paola Ghiorzo, Francesco Spagnolo

**Affiliations:** ^1^Medical Oncology, IRCCS Ospedale Policlinico San Martino, Genoa, Italy; ^2^Genetics of Rare Cancers, IRCCS Ospedale Policlinico San Martino, Genoa, Italy; ^3^Genetics of Rare Cancers, Department of Internal Medicine and Medical Specialties, University of Genoa, Genoa, Italy

**Keywords:** melanoma, *BRAF* mutation, targeted therapy, MAPK pathway, metastatic disease

## Abstract

Incidence of melanoma has been constantly growing during the last decades. Although most of the new diagnoses are represented by thin melanomas, the number of melanoma-related deaths in 2018 was 60,712 worldwide (Global Cancer Observatory, 2019). Until 2011, no systemic therapy showed to improve survival in patients with advanced or metastatic melanoma. At that time, standard of care was chemotherapy, with very limited results. The identification of *BRAF* V600 mutation, and the subsequent introduction of *BRAF* targeting drugs, radically changed the clinical practice and dramatically improved outcomes. In this review, we will retrace the development of molecular-target drugs and the current therapeutic scenario for patients with *BRAF* mutated melanoma, from the introduction of *BRAF* inhibitors as single agents to modern clinical practice. We will also discuss the resistance mechanisms identified so far, and the future therapeutic perspectives in *BRAF* mutated melanoma.

## Introduction

**FIGURE 1:**
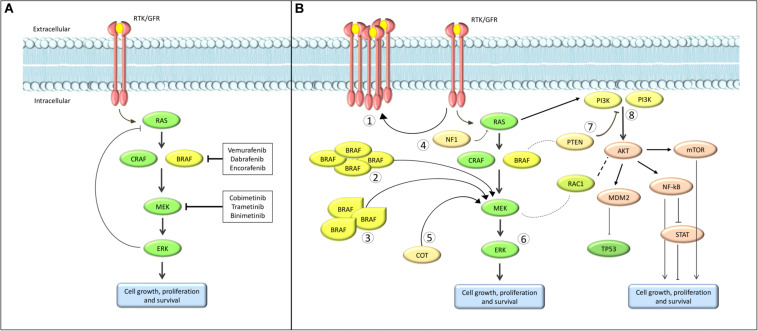
Schematic overview of the MAPK pathway. **(A)** normal pathway; **(B)** the most common resistance mechanisms. (1) Upregulation of RTK. (2) BRAF amplification. (3) BRAF alterantive splicing. (4) Loss of NF1. (5) COT overexpression. (6) ERK activation. (7) Loss of PTEN. (8) Alternative pathways activation.

Cutaneous melanoma has one of the highest mutational rate among all solid tumors (The Cancer Genome Atlas Network, 2015). Some of these mutations involve specific oncogenes, causing alterations in cell cycle regulation, proliferation and apoptosis. Multiple molecular pathways are implicated: among these, one of the most characterized is the Mitogen-Activated Protein Kinase (MAPK). This molecular pathway is composed by a Tyrosine Kinases Receptor (TKR), RAS, RAF, MEK and ERK proteins (Figure 1). Simplifying, the binding between a growth factor and the TKR leads to a phosphorylation cascade resulting in the activation of ERK. ERK, in turn, regulates the expression of many genes involved in cell proliferation and survival (Gaestel, 2006). The mutation of a gene coding for one of these proteins can constitutively activate the whole pathway.

Activating *BRAF* mutation occurs in approximately 50% of cutaneous melanoma (The Cancer Genome Atlas Network, 2015; Sanchez-Vega et al., 2018). To date, about 300 *BRAF* mutations have been characterized, the most common being the V600E (valine to glutamic acid; 70–88%) (Rubinstein et al., 2010; Lovly et al., 2012; Menzies et al., 2012). The identification and characterization of *BRAF* mutations led to the development of highly specific drugs which radically changed the therapeutic landscape of melanoma. Indeed, targeted therapies substantially improved survival in patients with advanced or metastatic melanoma from a median of 6 months obtained with chemotherapy (Korn et al., 2008), the standard of care before the approval of the first BRAF inhibitor, to a median of 25.9–33.6 months (Robert et al., 2019; Ascierto et al., 2020). Moreover, targeted therapies showed a significant benefit in the adjuvant setting with a 53% decrease in the risk of relapse compared with placebo (Long et al., 2017b). These results recently lead to the approval of BRAF plus MEK inhibitors for high risk resected (stage III) melanoma patients (Long et al., 2017b; Spagnolo et al., 2019). These revolutionary changes underline the importance of the early molecular characterization of high-risk stage II, stage III and IV melanoma patients, which has become mandatory according to the ESMO Clinical Practice Guidelines (Michielin et al., 2019) and represents a fundamental step for personalized therapy. For this reason, the assessment of *BRAF* mutations nowadays constitutes a fundamental diagnostic procedure and essential in the current clinical practice of oncology. The molecular biology-based strategies used for *BRAF* mutation detection have been extensively described in a related review (Vanni et al., 2020).

In this review we will retrace the development of molecular-target drugs and the current therapeutic scenario for patients with *BRAF* mutated melanoma, from the introduction of BRAF inhibitors as single agents in 2011 to modern clinical practice. We will also discuss the resistance mechanisms identified so far, and the future therapeutic perspectives in BRAF mutated melanoma.

### BRAF Inhibitors

The first drug used as BRAF inhibitor in patients with *BRAF* V600E advanced or metastatic melanoma was sorafenib (BAY 43-9006), which showed promising results in murine models but failed the human experimentation (Eisen et al., 2006; Hauschild et al., 2009).

In 2005 and later in 2009, BRAF inhibitors PLX4032 (vemurafenib) and GSK2118436 (dabrafenib) were synthesized. Finally, in 2013 LGX818, or encorafenib, began clinical investigation.

In the randomized phase 3 studies BRIM-3 (Chapman et al., 2011, 2017; McArthur et al., 2014) and BREAK-3 (Hauschild et al., 2012; Latimer et al., 2015), BRAF inhibitors vemurafenib and dabrafenib, respectively, obtained a statistically significant benefit in terms of overall survival (OS), progression-free survival (PFS) and overall response rate (ORR) compared to chemotherapy (Table 1).

**TABLE 1:** Summary of selected targeted therapy trials in BRAF-mutant advanced melanoma.

Trial	Drugs		Median OS (mo)	Median PFS (mo)	ORR	References
BRIM-3	Vemurafenib		13.6	6.9	57%	McArthur et al., 2014; Chapman et al., 2017
	Dacarbazine		9.7	1.6	9%	
BREAK-3	Dabrafenib		18.2	6.7	53%	Hauschild et al., 2012; Latimer et al., 2015
	Dacarbazine		15.6	2.9	6%	
Combi-D	Dabrafenib + trametinib		25.1	11	69%	Long et al., 2015
	Dabrafenib		18.7	8.8	53%	
Combi-V	Dabrafenib + trametinib		26.1	12.1	67%	Robert et al., 2016
	Vemurafenib		17.8	7.3	53%	
CoBRIM	Vemurafenib + Cobimetinib		22.3	12.3	68%	Society for Melanoma Research Congress, 2019
	Vemurafenib		17.4	7.2	50%	
COLUMBUS	Encorafenib + Binimetinib		33.6	14.9	64%	Ascierto et al., 2020
	Vemurafenib		16.9	7.3	41%	
	Encorafenib		23.5	9.6	52%	

**Trial**	**Drugs**	**Cohorts**	**Median OS**	**Median PFS**	Intracranial response (%) by investigators – IRC*	**References**

BREAK – MB	Dabrafenib	A (previously untreated BM)	33.1 weeks	16.1 weeks	39.2%** – 20% **	Long et al., 2012
		B (previously treated BM)	31.4 weeks	16.6 weeks	30.8%** – 19%**	
Vemurafenib	Vemurafenib	1 (previously untreated BM)	8.9 month	3.7 months	29% – 18%	Mcarthur et al., 2016
		2 (previously treated BM)	9.6 month	4 months	23% – 18%	
COMBI – MB	Dabrafenib + trametinib	A (BRAF V600E asymptomatic, previously untreated BM)	10.8	5.6	58%	Davies et al., 2017
		B (BRAF V600E asymptomatic, previously treated BM)	24.3	7.2	56%	
		C (BRAF V600D/K/R, asymptomatic, previously treated or untreated BM)	10.1	4.2	44%	
		D (BRAF V600D/E/K/R, symptomatic, previously treated or untreated BM)	11.5	5.5	59%	

* Independent review committee (IRC). ** Considering *BRAF* V600E-mutated patients. Abbreviations: BM, brain metastases; OS, overall survival, PFS, progression-free survival; ORR, overall response rate; IRC, independent review committee; MO, months.

These results fueled molecular targeted drug research and raised several new issues.

First, the problem of resistance. Indeed, about 15% of patients showed no response to BRAF inhibition (Spagnolo et al., 2015) and, among responders, about 50% developed acquired resistance after a median of 6–8 months.

Another challenge was the paradoxical effect of BRAF inhibitors on BRAF-wild type cells.

Safety analysis of BRIM-3 study showed that about 20% of patients developed cutaneous squamous carcinoma. This phenomenon is due to a collateral activation of the MAPK pathway in BRAF-wild type keratinocytes (Heidorn et al., 2010; Poulikakos et al., 2010; Su et al., 2012). The mechanism behind this paradoxical effect is not entirely clear but it seems related to conformational change in wild-type BRAF protein, CRAF dimerization and ERK activation. This process might also induce new malignant melanoma (Zimmer et al., 2012; Dalle et al., 2013), RAS-mutant leukemia (Callahan et al., 2012) and other second neoplasms (Gibney et al., 2013).

### BRAF Inhibitors Plus MEK Inhibitors

Preclinical studies suggested that the addition of a MEK inhibitor to a BRAF inhibitor could reduce the side effects of BRAF inhibitor as single agent (e.g., paradoxical effect), delay the development of resistance and generate a synergistic improvement in efficacy outcomes (King et al., 2013). On this wave, several studies have been performed. Taken together (Pasquali et al., 2018) data from these clinical trials demonstrated a statistically significant superiority of the combination compared to monotherapy in terms of OS, PFS and ORR (Queirolo and Spagnolo, 2017; Table 1).

*Dabrafenib plus Trametinib*. The phase I/II trial (Flaherty et al., 2012) demonstrated the safety of dabrafenib and trametinib combination and its significant superiority in terms of ORR and PFS over dabrafenib monotherapy, among patients with *BRAF* V600E/K-mutated, unresectable or metastatic melanoma. Moreover, a subsequent survival analysis showed its advantage in terms of survival (Long et al., 2016). Based on these results, the combination of dabrafenib and trametinib received FDA approval in January 2014, with an accelerated procedure.

The phase III trial, COMBI-d (Long et al., 2014) compared dabrafenib plus trametinib with dabrafenib monotherapy. In the primary analysis, median PFS and ORR were significantly increased in the combination arm. A subsequent update (Long et al., 2015) demonstrated that the combination reduced the risk of death by 29% compared with monotherapy with a 3 years OS of 44 vs 32% (Long et al., 2017a).

Combination of dabrafenib and trametinib was also tested in another phase III trial, the COMBI-v study (Robert et al., 2015), in comparison with vemurafenib. Data showed a significant benefit for the combination in terms of ORR, median PFS and median OS. Even better results were obtained in the population with normal lactate dehydrogenase (LDH), in terms of median PFS [17.5 vs 9.2 months – Hazard Ratio (HR) 0.55] and survival (median OS not reached vs 21.5 months – HR 0.56) (Robert et al., 2016). Similar results, in terms of clinical activity, were observed in real world population, with an ORR of 67% (Atkinson et al., 2020).

The most recent update of these studies is a pooled analysis published in 2019 (Robert et al., 2019). Efficacy outcomes were confirmed to be completely superimposable between the two trials, underling the strength of the evidence, and showed a 5-year PFS of 19% and a 5-year OS of 34%.

*Vemurafenib plus Cobimetinib*. In the Phase Ib study BRIM-7 (Ribas et al., 2014), vemurafenib plus cobimetinib showed a significant benefit in terms of ORR, PFS and OS, and the results of the subsequent phase III trial, coBRIM (Larkin et al., 2014), led to the FDA registration of this combination. Indeed, combination therapy showed its superiority on the comparison arm (vemurafenib) in terms of both PFS (HR 0.51) and ORR. A subsequent analysis (Society for Melanoma Research 2016 Congress, 2017) showed a higher number of complete response (16 vs 11%), which suggested that some patients may get a better response if treatment is continued. Final analysis has been presented at Society for Melanoma Research (SMR) congress 2019 (Society for Melanoma Research Congress, 2019), and confirmed the substantial superiority of the combination over monotherapy.

*Encorafenib plus Binimetinib*. In the phase III study COLUMBUS (Dummer et al., 2018), 577 patients received encorafenib (450 mg daily) plus binimetinib (45 mg twice daily) or encorafenib (300 mg) or vemurafenib (960 mg twice daily). The study was divided into two parts: the first one aimed to compare efficacy of the combination vs vemurafenib (primary endopoint) and encorafenib, while the second one aimed to characterize the contribute of binimetinib in the obtained outcome.

Results of part one showed the superiority of encorafenib plus binimetinib combination over vemurafenib in terms of ORR, PFS and OS. Analysis at 3 years showed an OS of 47% for the combination, 41% for encorafenib and 31% for vemurafenib, with a death risk reduction of 39% (Ascierto et al., 2020). Also in this case, a recently published landmark analysis showed that the subgroups of patients with normal LDH and less than three metastatic sites were the ones with the best outcomes (Ascierto et al., 2020).

Part 2 of the study randomized 344 patients in a 3:1 ratio to receive either encorafenib 300 mg plus binimetinib 45 mg twice daily or encorafenib 300 mg. Results, presented at ESMO 2017 (Dummer et al., 2017), confirmed the superiority of combination treatment over monotherapy in terms of ORR, PFS and OS.

*Safety.* Globally, combination therapies are well tolerated and present an acceptable toxicity profile. Some adverse events are quite common in all combination schemes, such nausea, diarrhea, vomiting, fatigue, headache, arthralgia and a lower rate of cutaneous squamous cell carcinomas. Other adverse events are more frequent with specific combination, like pyrexia and chills for dabrafenib and trametinib, photosensitivity and diarrhea for vemurafenib and cobimetinib and laboratory alterations with encorafenib and binimetinib. Finally, dabrafenib and trametinib showed to significantly improve patients’ quality of life compared to both dabrafenib and vemurafenib monotherapy (Grob et al., 2015; Schadendorf et al., 2015), while vemurafenib and cobimetinib showed to maintain the patient’s quality of life compared with vemurafenib monotherapy, increasing efficacy (Dréno et al., 2018).

### Brain Metastasis

Brain involvement is frequent in melanoma patients and is associated with poor prognosis (Davies et al., 2011; Spagnolo et al., 2016). About 20% of patients have brain metastases (BM) at the initial diagnosis of advanced disease, and more than 40% develop BM at some point of their disease course; up to 75% patients have BM in autopsy studies (Long and Margolin, 2020). Before the introduction of immunotherapy and target therapy, median OS of this group of patients was 3.8–5.0 months (Davies et al., 2011).

*BRAF inhibitors*. Dabrafenib and vemurafenib were tested through two phase II trial. In both studies patients were divided into two cohorts: one cohort included patients naive for previous BM local treatment, while the second cohort included patients who progressed after BM local treatment (surgery, WBRT, or SRS). Both dabrafenib (BREAK-MB) (Long et al., 2012) and vemurafenib (Mcarthur et al., 2016) showed clinical activity in both cohorts in terms of intracranial ORR, PFS and OS (Table 1). Of note, dabrafenib obtained greater results among *BRAF* V600E-mutated than *BRAF* V600K-mutated patients. In both trials, the extracranial OR, the median DOR, the median PFS and OS were lower than that observed in previous studies with dabrafenib and vemurafenib, and these data support the hypothesis that BM in *BRAF*-mutant melanoma are less responsive to BRAF inhibition, probably due to some different characteristics of BM, or differences in drug concentrations between intracranial and extracranial compartments.

*Combination of BRAF plus MEK inhibitors*. In COMBI-MB, a phase II study, 125 *BRAF* V600-mutated melanoma patients with BM were divided into four cohorts and treated with dabrafenib plus trametinib (Davies et al., 2017). Cohort A included 76 *BRAF* V600E-mutated, asymptomatic patients, naive for local brain therapy; cohort B included 16 *BRAF* V600E-mutated, asymptomatic patients, previously treated with local brain therapy; Cohort C included 16 *BRAF* V600D/K/R-mutated, asymptomatic patients, naive for local brain therapy or previously treated; Cohort D included 17 *BRAF* V600D/E/K/R-mutated, symptomatic patients, naive for local brain therapy or previously treated. The primary endpoint of the trial, the intracranial response in cohort A, was met with a rate of 58% including a 4% of intracranial complete response, and a median duration of response of 6.5 months. Intracranial response was also seen in cohorts B, C and D but these data should be considered exploratory, due to the sample size of the two cohorts. Response rate was lower than observed in patients with just extracranial disease (58 vs 67%) and the median PFS was significantly shorter (5.6 vs 10.1 months), which suggests an earlier treatment failure in BM. Finally, another clinical trial with vemurafenib, cobimetinib and atezolizumab is currently ongoing and results are awaited (NCT03625141) (ClinicalTrials.gov, 2020).

### Adjuvant Setting

Considering the extraordinary results obtained in the metastatic setting, studies on the efficacy of BRAF and MEK inhibitors in the adjuvant setting were initiated (Long et al., 2017b; Spagnolo et al., 2019).

In the BRIM-8 trial (Maio et al., 2018), patients diagnosed with stage IIC, IIIA, IIIB and IIIC *BRAF*-mutated melanoma were randomized to receive either vemurafenib or placebo. Patients were divided into two cohorts: cohort one included stage IIC, IIIA, IIIB, and cohort two included patients with IIIC disease. Results of the study showed that 1 year of adjuvant vemurafenib provided a substantial disease-free survival (DFS) benefit (46% risk reduction vs placebo) in cohort one, while in cohort two increased median DFS, demonstrating a biologic effect, but did not statistically significantly reduce DFS risk.

In the phase III COMBI-AD trial (Long et al., 2017b), 870 patients diagnosed with IIIA (with lymph node metastasis >1 mm), IIIB and IIIC *BRAF*-mutated melanoma were treated with dabrafenib plus trametinib or placebo for 1 year. Primary endpoint was relapse-free survival (RFS), while OS and distant metastasis-free survival (DMFS) were exploratory endpoints. In October 2018, results with about 4 years of follow-up were published. Data showed a 4-year RFS of 54% in the experimental arm vs 38% in the placebo arm (HR 0.49) with an estimated cure rate of 54 vs 37%, respectively (Hauschild et al., 2018). Three-year OS was 86 vs 77%. Notably, the delta between the curves increased over time, suggesting a potential long-term impact on survival.

### Resistance and Beyond

During treatment with BRAF plus MEK inhibitors, primary and acquired resistance remain a significant challenge (Figure 1). Several preclinical studies tried to understand mechanisms of resistance, with the aim of preventing or blocking them. Simplifying, it is possible to divide the most understood mechanisms of resistance between intracellular and extracellular mechanisms.

Among intracellular mechanisms, the most characterized include the reactivation of the MAPK signaling pathway with ERK activation (Nissan et al., 2013; González-Cao et al., 2015) or overexpression of TKR, and the activation of alternative intracellular molecular pathways (Figure 1).

Several ERK-inhibitors are currently in development (Carlino et al., 2014). GDC-0994, a highly selective ERK1/2 inhibitor, has demonstrated activity in combination with cobimetinib in preclinical models (Robarge et al., 2014). SCH772984 has shown promising results in a panel of melanoma cell lines, including cells with innate or acquired resistance to vemurafenib, cells with *BRAF/NRAS* double mutations or *NRAS* mutations (Morris et al., 2013; Wong et al., 2014) (NCT02457793).

Blocking the MAPK pathway at the RTK level has a strong rationale, if we consider that RTK could activate simultaneously MAPK and alternative pathways like PI3K-AKT-mTOR (Nazarian et al., 2010; Villanueva et al., 2010; Straussman et al., 2012; Wilson et al., 2012): multi-RTK-inhibitors (e.g., lenvatinib), selective small-molecule RTK-inhibitors (capmatinib, BGJ398, and MEHD7945A), and monoclonal antibodies that bind the extracellular domain of the RTK (onartuzumab, ganitumab) are in course of study in combination with BRAF- or MEK-inhibitors (ClinicalTrials.gov, 2020). Moreover, numerous inhibitors that target various levels of the PI3K pathway are in development, including PI3K, AKT, and mTOR inhibitors (Shi et al., 2014; Manzano et al., 2016).

Regarding extracellular mechanisms, increasingly strong arguments support the importance of the tumor microenvironment and the modulation of the immune system.

These observations led to the develop of immune-checkpoints inhibitors, such ipilimumab, nivolumab, and pembrolizumab, monoclonal antibodies aimed to interact with CTLA-4 and PD1 to re-establish immune response against the tumor. All these molecules allowed to obtain durable responses and long-term survival in patients with advanced or metastatic melanoma. At the same time, several studies demonstrated that BRAF inhibitors can impact on immune responses in a direct (e.g., increase of intratumour infiltrating lymphocytes – TILs) and an indirect (increase of melanoma antigens expression) way suggesting a strong rationale of combining these two therapeutic strategies (Boni et al., 2010; Khalili et al., 2012; Koya et al., 2012; Frederick et al., 2013; Sapkota et al., 2013; Kakavand et al., 2015).

The first combination trials with ipilimumab and vemurafenib unfortunately demonstrated significant toxicities that limited further development (Ribas et al., 2013; Minor et al., 2015).

Combination strategies with BRAF inhibitors and anti–PD-1/PD-L1 agents have also been tested.

First, the combination of vemurafenib plus atezolizumab (anti–PD-L1). Early data from this experimentation were presented at the SMR 2015 Congress and showed an ORR rate of 76%, without unexpected adverse events being reported. Encouraged by these exciting results, this trial was expanded adding cobimetinib to vemurafenib and atezolizumab, reaching an ORR of 83% (Society for Melanoma Research 2016 Congress, 2017). Based on these findings, a randomized phase III study with vemurafenib, cobimetinib and atezolizumab/placebo was initiated, but results are not available yet (NCT02908672) (ClinicalTrials.gov, 2020).

A similar approach with dabrafenib, trametinib and pembrolizumab was tested in the phase I (Ribas et al., 2016) and II trial KEYNOTE-022 (NCT02130466) (ClinicalTrials.gov, 2020), that compared the triple combination vs dabrafenib, trametinib and placebo. Data from the phase II part, presented during SMR Congress 2019 (Society for Melanoma Research Congress, 2019), with a median follow-up of 28 months, showed a median PFS (primary endpoint) of 16.9 months for the pembrolizumab plus dabrafenib and trametinib arm vs 10.7 months for placebo plus dabrafenib and trametinib arm (HR 0.53) with a 24-months PFS rates of 41 and 16.3%. Unfortunately, these results were not statistically significant and the primary endpoint of the study was not met. Median OS in the triple combination arm was not reached vs 26.3 months in dabrafenib plus trametinib arm (HR 0.64), and OS rates at 24-months were 63 and 51.7% respectively. Moreover, the duration of response was longer in the experimental arm.

Finally, acquired resistance could be also caused by epigenetic changes that may be reversible (Van Allen et al., 2014). For this reason, another interesting approach in melanoma patients is the rechallenge with targeted therapies, despite data from randomized trials are lacking. In a recently published minireview (Reschke et al., 2019), 238 patients from several retrospective and prospective trials were analyzed showing a disease control rate of 67%.

## Conclusion

In summary, targeted therapy with BRAF plus MEK inhibitors has radically changed the therapeutic landscape of melanoma, both in the advanced and the adjuvant setting. In the absence of a standardized therapeutic algorithm for BRAF mutated patients, clinicians can choose whether to start with BRAF plus MEK inhibitors or with immunotherapy, on the basis of the experience of their center, characteristics of the patient (i.e., his compliance with treatment, concomitant pathologies), and characteristics of the disease (i.e., tumor burden, LDH level).

Despite the many advances made in the therapy of these patients and the exciting results achieved, some issues remain unanswered.

Among all, one the most important is the identification and overcoming of primary and acquired resistances. Numerous drugs are currently being tested attempting to extend the pharmacological inhibition beyond MAPK pathway targeting parallel pathways molecules. Moreover, having ascertained that some forms of resistance involve epigenetic and transient mechanisms, the rechallenge with BRAF plus MEK inhibitors in resistant patients, progressing after a subsequent line (e.g., immunotherapy), is being further investigated. Furthermore, the data from clinical trials with combinations of targeted drugs and immunotherapy will be extremely interesting, especially in patients with complex clinical situations such as elevated baseline LDH and brain metastases.

Lastly, an important issue that needs further investigation is the treatment beyond progression, a therapeutic strategy that allows to continue the same systemic treatment in case of local progression, which could be managed with local treatments such as surgery or radiotherapy, providing that patient has a good Eastern Cooperative Oncology Group (ECOG) Performance Status and good tolerance to targeted therapy. This therapeutic strategy, commonly accepted in other solid cancers (Kuczynski et al., 2013), is still debated in melanoma. However, a retrospective study published in 2014 suggested that it may be appropriate to consider treatment beyond progression, postponing the start of a new line of therapy without evident detrimental effects (Chan et al., 2014). Similar results were achieved in another retrospective series (Scholtens et al., 2015). Currently, a study promoted by the Italian Melanoma Intergroup (IMI) aimed to prospectively assess the clinical impact of treatment beyond progression with vemurafenib plus cobimetinib, is recruiting patients in 12 Italian Centers (NCT03514901) (ClinicalTrials.gov, 2020).

This consideration underlines the importance of proceeding with both vertical and horizontal inhibition, blocking MAPK pathway but also other molecular pathways like PI3K/mTOR.

## Author Contributions

ET and IV contributed equally in conceiving the review focus, conducting the literature review, summarizing the manuscript, writing the first draft, and finalizing the manuscript. PG and FS designed and directed the review. AB, VA, WB, PG, and FS revised and made corrections to the manuscript. All authors have read and agreed to the final version of the manuscript.

## Conflict of Interest

The authors declare that the research was conducted in the absence of any commercial or financial relationships that could be construed as a potential conflict of interest.
